# Targets for Ibrutinib Beyond B Cell Malignancies

**DOI:** 10.1111/sji.12333

**Published:** 2015-08-18

**Authors:** A. Berglöf, A. Hamasy, S. Meinke, M. Palma, A. Krstic, R. Månsson, E. Kimby, A. Österborg, C. I. E. Smith

**Affiliations:** ^1^ Department of Laboratory MedicineKarolinska InstitutetStockholmSweden; ^2^ Center for Hematology and Regenerative MedicineKarolinska Institutetand Department of Clinical Immunology and Transfusion MedicineKarolinska University Hospital HuddingeStockholmSweden; ^3^ Department of HematologyKarolinska University Hospital SolnaStockholmSweden; ^4^ Department of Oncology and PathologyKarolinska InstitutetStockholmSweden; ^5^ Center for Hematology and Regenerative MedicineKarolinska InstitutetStockholmSweden; ^6^ Department of MedicineKarolinska InstitutetStockholmSweden

## Abstract

Ibrutinib (Imbruvica^™^) is an irreversible, potent inhibitor of Bruton's tyrosine kinase (BTK). Over the last few years, ibrutinib has developed from a promising drug candidate to being approved by FDA for the treatment of three B cell malignancies, a truly remarkable feat. Few, if any medicines are monospecific and ibrutinib is no exception; already during ibrutinib's initial characterization, it was found that it could bind also to other kinases. In this review, we discuss the implications of such interactions, which go beyond the selective effect on BTK in B cell malignancies. In certain cases, the outcome of ibrutinib treatment likely results from the combined inhibition of BTK and other kinases, causing additive or synergistic, effects. Conversely, there are also examples when the clinical outcome seems unrelated to inhibition of BTK. Thus, more specifically, adverse effects such as enhanced bleeding or arrhythmias could potentially be explained by different interactions. We also predict that during long‐term treatment bone homoeostasis might be affected due to the inhibition of osteoclasts. Moreover, the binding of ibrutinib to molecular targets other than BTK or effects on cells other than B cell‐derived malignancies could be beneficial and result in new indications for clinical applications.

## Introduction

Ibrutinib is a potent, orally available Bruton's tyrosine kinase (BTK)^1^ inhibitor belonging to a class of therapeutics termed ‘targeted covalent drugs’ and demonstrates promising preclinical and clinical activity in several B cell malignancies 1, 2. BTK is a non‐receptor tyrosine kinase that belongs to the TEC family kinases (TFK), which is the second largest family of non‐receptor kinases in humans 3. BTK is known to be an essential component of B cell receptor (BCR) signalling and is involved in B cell differentiation, proliferation and survival 4, 5. Many studies have reported an essential role of BCR signalling in the pathogenesis of several B cell malignancies 6. As a key molecule for this pathway, BTK became an important target for the treatment of B cell‐derived tumours.

Ibrutinib inactivates BTK by binding covalently to Cysteine 481 in the ATP‐binding site in an irreversible manner 7. This active site occupancy inhibits the subsequent phosphorylation of BTK, phospholipase C*γ*2 (PLC*γ*2), AKT and ERK and abolishes the BCR signalling downstream of BTK both *in vitro* and *in vivo* 8. Furthermore, inhibition of BTK impairs proliferation, survival and induces apoptosis of malignant B cells 9.

Thus, many preclinical and clinical studies have shown a profound antitumour activity of ibrutinib in different B cell malignancies, including chronic lymphocytic leukaemia (CLL), mantle cell lymphoma (MCL), multiple myeloma, diffuse large B cell lymphoma (DLBCL) and Waldenstrӧm's macroglobulinemia (WM) 10, 11, 12.

Ibrutinib (Imbruvica^™^; Pharmacyclics, Inc., Sunnyvale, CA, USA) received approval from the U.S. Pharmacyclics, Inc. 999 East Arques Avenue Sunnyvale, California 94085 Food and Drug Administration (FDA) for MCL in November 2013, for CLL in July 2014 and for WM in January 2015 1, 12, 13. The use of the drug has also been approved by the European Medicines Agency (EMA) for the treatment of CLL and MCL. Ongoing studies are also evaluating ibrutinib as a therapy for other B cell malignancies as well as inflammatory and autoimmune diseases, as the results in animal models provided strong evidence of its effectiveness 14, 15.

**Figure 1:**
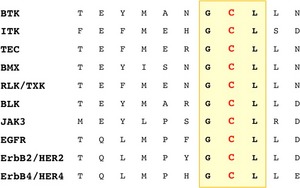
Alignment of kinases having a cysteine residue in the ATP‐binding site corresponding to cysteine 481 in Bruton's tyrosine kinase.

Kinase selectivity is an important issue for small molecule inhibitors affecting their efficacy and safety. Ibrutinib exhibits remarkable selectivity for BTK. However, nine other kinases have a corresponding cysteine residue in the ATP‐binding site. These include four TFK members (ITK, TEC, BMX and RLK/TXK), three EGFR family kinases (EGFR, ErbB2/HER2 and ErbB4/HER4) and two other kinases, BLK and JAK3 2 (Fig. 1). Ibrutinib shows different affinity for these kinases (Table 1). Even among TFKs, it shows differential binding. The half maximal inhibitory concentration (IC_50_) of ibrutinib for inhibition of BTK enzymatic activity is 0.5 nm, while the corresponding value for the weakest binder TEC, is 78 nm. It has also been shown by *in vitro* assays that ibrutinib binds to and inhibits other proteins that lack the cysteine residue. The binding of the drug to these kinases is reversible. However, it is of importance that the affinity of binding for the proteins such as BRK, CSK, FRG and HCK is in the low nanomolar range, that is not very different from that of BTK 15. Another molecule with high binding affinity (IC_50_ 0.5 nm) and ibrutinib binding site is BLK (Table 1). Interestingly, this protein was recently reported to be involved in regulation of pro‐inflammatory cytokine production and its reduced activity could contribute to the development and pathogenesis of autoimmune disorders 16.

**Table 1:** The expression and biological functions of proteins with ibrutinib binding sites

Proteins	Expression	Biological function	Binding of ibrutinib IC_50_ (nm) 15
TEC family kinases			
BTK	B cells, platelets, erythrocytes, macrophages, neutrophils, mast cells, dendritic cells, NK cells 27, 28	TEC family kinases, non‐receptor tyrosine kinases playing an important role in signalling pathways in hematopoietic cells 29	0.5
TEC	B cells, T cells, platelets, erythrocytes, macrophages, neutrophils, mast cells, liver, heart 27, 29, 30		78
ITK	T cells, NK cells, mast cells 27		10.7
BMX	Macrophages, neutrophils, endothelial cells, arterial endothelium 27, 31		0.8
RLK/TXK	T cells, NK cells, mast cells 27, 29, 32		Not reported
Other kinases			
BLK	B cells, thymocytes, plasmacytoid dendritic cells 16, 33	Non‐receptor tyrosine kinase of the SRC‐family kinases, involved in B‐lymphocyte development, differentiation and signalling 16	0.5
EGFR	Epithelial cells 34	Epidermal growth factor receptor (EGFR) family of receptor tyrosine kinases involved in cell growth, proliferation and differentiation 34	5.6
ErbB2/HER2			9.4
ErbB4/HER4			Not reported
JAK3	B cells, T cells, NK cells, myeloid cells, vascular smooth muscle cells, endothelium 35	Janus family of kinases, involved in cytokine receptor‐mediated intracellular signal transduction, cell proliferation and differentiation 35	16.1

Ibrutinib is generally well tolerated, and long‐term therapy with ibrutinib is associated with modest toxicity 17. The majority of the adverse events are grade 1 or 2 in severity and typically resolving without additional therapy 18. The most common adverse events are diarrhoea, fatigue, bleeding and infections 17, 19. Some of these events are off‐target‐related adverse effects. For instance, recent studies on human platelets suggested that the increased risk of bleeding associated with ibrutinib treatment was due to platelet dysfunction caused by inhibition of both BTK and TEC 20.

The off‐target effects of ibrutinib on cells inside as well as outside the haematopoietic system could be useful in treatment of other diseases. In HER2‐positive breast cancer, the aberrant expression of HER2 is associated with poor prognosis of the disease. A recent study showed that ibrutinib is an effective inhibitor of HER2 and that treatment with ibrutinib completely abrogated the phosphorylation of HER2 and potently inhibited the cell viability of HER2‐positive breast cancer cells 21. Ibrutinib treatment also leads to dose‐dependent inhibition of EGFR phosphorylation and it is speculated that it could be a candidate drug for the treatment of EGFR‐mutant, non‐small cell lung cancer 22. Other studies show that ibrutinib is an effective mast cell inhibitor and treatment of insulinoma‐bearing mice with this drug blocks mast cell degranulation and triggers collapse of tumour vasculature, tumour hypoxia and regression 23. In a preclinical model of pancreatic ductal adenocarcinoma as well as in patient‐derived xenograft‐models, ibrutinib showed mast cell dependent, antifibrotic activity and improved survival 24. Based on this finding, it was also suggested that the use of ibrutinib could potentially be extended to the treatment of other fibrotic diseases or chronic pancreatitis. Furthermore, other studies have shown that ibrutinib can inhibit inflammasome activation by BTK and TEC and attenuate inflammatory responses. Inhibition of TEC in a murine model of fungal infection protected the treated mice from fungal sepsis suggesting the drug could be used to combat invasive microbial infections 25. Targeting BTK could also have a therapeutic potential in ischaemic stroke, as ibrutinib suppresses the inflammasome in the ischaemic brain 26.

## TEC family kinases

TFKs are expressed preferentially in different lineages of haematopoietic cells but also in other organs, such as liver (TEC) or in endothelial cells (BMX), as previously reviewed 29 and as summarized in Table 1. Loss‐of‐function mutations of the family members BTK and ITK, as well as elevated expression of TXK and ITK are associated with human disease. All members of TFKs have been knocked out in mice and their phenotypes have been studied, as summarized in Table 2. There, we focus on B and T cell compartments and compare KO mouse phenotypes with human diseases related to the dysfunction of corresponding genes.

**Table 2:** TEC family kinases: the human diseases and the phenotype of KO mice

Proteins	Human disease	Knock‐out mouse phenotype (B and T lymphocyte compartments)
BTK	X‐linked agammaglobulinemia: Lack of mature B cells and gammaglobulin production (<1% CD19^+^ cells in peripheral blood) 36, 37	X‐linked immunodeficient (XID) and BTK KO mouse: milder B cell deficiency than in XLA patients with 50% of the peripheral B cells remaining, loss of B1 B cells in the peritoneal cavity, immature phenotype of peripheral B cells, low levels of secretory IgM and IgG3, absent responses to T cell independent type II antigens 39, 40
TEC	Not reported	No overt B cell phenotype 42
		Tec KO mouse: enhanced generation of CD44^high^CD62L^−^ Th17 subset of CD4^+^ T cells 43
ITK	ITK mutation: EBV‐associated lymphoproliferative disorder 44, 45	ITK KO mouse: increased percentage of memory phenotype CD44^high^CD62L^−^CD4^+^ T cells and CD8^+^ T cells of almost exclusively CD44^high^CD62L^+^ innate‐like phenotype 46, 58, defective Th2 and Th17 differentiation and function, increase in regulatory T cell (Treg) numbers and function 50, 51, defective NKT 59
	ITK increased expression: patients with atopic dermatitis 49	
BMX	Not reported	No overt phenotype 53
RLK/TXK	TXK increased expression: patients with Behcet's disease 56	No overt phenotype, marginal T cell defect 60

Thus, mutations in the *BTK* gene in humans cause a severe B cell deficiency X‐linked, agammaglobulinemia (XLA) 36, 37. In affected individuals, a defect in B cell maturation and function leads to the profound reduction of serum immunoglobulins and increased susceptibility to infections 38. A less severe, B cell disorder manifested at later stages of B cell development is observed in mice carrying BTK mutations 39, 40. It seems that in mice TEC partially compensates for the loss of BTK, while this does not occur in humans, suggesting that the corresponding signal transduction pathways are not identical in these species (Table 3). Of interest is also the fact that BTK seems to be required for NK cell activation and cytokine production 28, 41. Surprisingly, patients treated with ibrutinib do not develop an XLA‐like disease and the immunoglobulin (Ig) blood levels in patients receiving this drug were not reduced even after 12 cycles (35‐day cycle) of ibrutinib, suggesting that drug activity can be limited to immature B cells 18 or that antibody levels are maintained due to the fact that Ig producing plasma cells are not BTK dependent. However, in the mouse models for rheumatoid arthritis and systemic lupus erythematosus, the production of autoantibodies was reduced after ibrutinib treatment 14, 15. This suggests that ibrutinib would influence antibody production towards immunogens acquired during the treatment.

TEC does not appear to be crucial for B cell maturation, as TEC KO mice do not have any altered phenotype in the B cell compartment 42. However, other studies show that TEC deficiency leads to the enhanced generation of Th17 effector/memory subsets *in vivo*. Moreover, CD44^high^CD62L^−^CD4^+^ T cells are slightly increased in TEC KO mice, and they produce more IL‐17 upon activation when compared to WT mice 43. ITK mutations in humans have been reported in several cases of a fatal Epstein–Barr virus (EBV)‐associated lymphoproliferative disorder 44, 45. ITK KO mice show altered T cell development and mature T cell effector function, affecting in particular conventional T cells, thus leading to increased numbers of CD4^+^ T cells with a CD44^high^CD62L^−^ memory phenotype and CD8^+^ T cells, with a CD44^high^CD62L^+^ innate‐like phenotype, that upon stimulation rapidly secrete high levels of effector cytokines such as IFN‐*γ* 46. The Th2 deficiency related to the lack of the ITK function, has made this kinase an interesting therapeutic target in asthma as ITK KO mice are resistant to ovalbumin‐induced asthma 47, 48. Furthermore, studies in 59 patients with atopic dermatitis, which is a Th2 mediated disease, showed highly elevated levels of ITK in peripheral blood T lymphocytes 49. It was also shown that ITK deletion affects the function of Th17 cells and that there is an increase in regulatory T cell (Treg) numbers and function in the absence of this kinase. This makes ITK a promising drug target for autoimmune disorders 50, 51. It was also found that in human NK cells ITK plays a role in regulating cell‐mediated cytotoxicity 52.

Human disease related to mutations in the *BMX* gene has not been reported as yet and the BMX KO mouse does not show an overt phenotype. Nonetheless, studies in the BMX KO mice suggest that this tyrosine kinase is involved in ischaemia‐induced arteriogenesis and angiogenesis 53. In addition to the role of BMX in the ischaemic response, BMX KO mice show reduced cardiac hypertrophy in a model of transverse aortic constriction, suggesting that BMX is required for the morphological response to pressure overload in the heart 54. Furthermore, it was shown that BMX participates in the wound healing process as observed in BMX transgenic mice 55.

Mutations affecting TXK have not been reported in humans as yet. TXK is preferentially expressed in Th1 cells. Its elevated expression was observed in patients with Behcet's disease, an inflammatory disorder associated with Th1 cytokine production and inflammation 56, 57, 58.

Many cell types express more than one TFK (Table 1) and loss‐of‐function mutants show additive functions. Mice deficient for various members of TEC family kinases give an important insight into the function of these proteins in different cell types and reveal biological processes for which these molecules are essential. It has been demonstrated for several of the tyrosine kinases that combined deletions (double KO mice) result in more severe phenotypes when compared to individual gene disruptions (Table 3). This can be exemplified by concomitant absence of BTK and TEC in B cells 42, ITK and TXK in both T cells 61 and NKT cells 59.

The dependence on more than a single TEC family kinase was also reported for BTK and TEC in macrophages 62, 63, osteoclasts 64 and platelets 65 (Table 3). Interestingly, compensatory pathways were described for BTK and TEC as well as for BTK and ITK in mast cells 66, 67, which will be described in more detail in the next sections of this review.

Even if multiple mutations affecting different TFK genes are unlikely to occur in the same individual, there is a risk that drugs binding to several target proteins and blocking their function could mediate such an outcome in treated patients. Here, studies in animals simultaneously defective in multiple TFKs would be of great value as demonstrated for SRC‐family protein kinases. There, only the BLK, FYN and LYN triple‐deficient mice have shown the profound impairment of the B cell development 68.

Thus, blocking the function of other/multiple TFKs would lead to ibrutinib off‐BTK target effects, as discussed below. Importantly, such effects could have either positive treatment outcomes, for example irreversible binding to ITK in T cells has been suggested as a treatment strategy for T cell malignancies 69 or negative, for example ibrutinib binding to both BTK and TEC in platelets could potentially lead to impaired coagulation and bleedings as observed in a subgroup of ibrutinib‐treated patients 20 (Table 3).

**Table 3:** The severity of the phenotype in mice defective for multiple TFKs

Cell type	More severe double KO phenotype when compared to single KO mice
B lymphocytes	BTK/TEC 42
T lymphocytes	ITK/TXK 59, 61
Platelets	BTK/TEC 65
Osteoclasts	BTK/TEC 64
Macrophages	BTK/TEC 62, 63
Mast cells	BTK/TEC, BTK/ITK 66, 67
Dendritic cells	Not reported
Erythroblasts/Erythrocytes	Not reported
Neutrophils	Not reported

## Consequences of ibrutinib treatment not related to B cells

### Ibrutinib affects ITK in T cells and NK cells

ITK is highly expressed in T cells. Upon T cell receptor (TCR) ligation ITK and RLK/TXK activate a signal cascade ultimately leading to cellular activation, proliferation and cytokine secretion 58. In CD8^+^ and Th1 CD4^+^ T cells, ITK plays a non‐essential supportive role to RLK/TXK. However, ITK retains a singular dominant role for the function of Th2 CD4^+^ T cells 50, 57, which do not express RLK/TXK 70 and for this reason, loss of ITK function impairs Th2 immunity.

Despite the fact that ITK shares significant sequence and functional homology with BTK, the possibility that it could be targeted by ibrutinib had been initially neglected due to a number of *in vitro* observations 15. Nevertheless, other *in vitro* observations pointed out that ibrutinib could affect T cell functions, for example production of certain cytokines 71.

In 2013, it was finally confirmed 69 that ibrutinib is capable to irreversibly bind to endogenous ITK in a T cell leukaemia cell line and in PBMC samples obtained from ibrutinib‐treated CLL patients. In the latter, the percentage of ITK bound to ibrutinib after 8 days of the treatment was found to be 40–80%, which was in line with *in vitro* data.

Furthermore, it was shown that ibrutinib could inhibit TCR‐induced ITK signalling and activation in both primary CD4^+^ and in Jurkat T cells as well as in CD4^+^ T cells collected from CLL patients receiving ibrutinib. Such inhibition neither affected TCR‐mediated cell proliferation nor T cell subsets (naïve, central, effector, and terminal memory). However, ibrutinib treatment affected T helper cell polarization as seen in the decrease of serum Th2‐type cytokines IL‐10, IL‐4 and IL‐13 as well as increase of IFN‐*γ* levels in CLL patients after 28 days of treatment. Accordingly, ibrutinib favoured Th1 polarization of human CD4^+^ cells *in vitro* and induced Th1/Th2 skewing of Ig subclasses in treated mice 69. It was concluded that ibrutinib selectively inhibits Th2 cells, because RLK/TXK is not expressed in these cells and cannot compensate for the ITK inhibited by ibrutinib 70. Nevertheless, the fact that ibrutinib seems to not bind to RLK/TXK is somewhat unexpected, as both kinases have ibrutinib binding sites. There is a possibility that the structure of RLK/TXK makes this protein less accessible for the drug. Still, this would need to be further investigated.

It is well known that one of the immune escape mechanisms exploited by tumours and by some pathogens is the subversion of adaptive immunity by promoting a Th2‐dominant helper T cell response favouring antibody production. This, in turn, has a detrimental effect on the ability of the immune system to trigger robust Th1 and CD8^+^ responses inducing direct effector cell cytotoxicity, thereby suppressing antitumour immune responses 72, 73.

The ability of ibrutinib to target ITK and therefore to modulate T cell responses suggested the possibility to combine this drug with other immune modulating therapies such as checkpoint inhibitors, that is agents interfering with either the CD80/CTLA‐4 or the PD‐1/PD‐L1 axis. Recently, the combination of ibrutinib with anti‐PD‐L1 antibody was tested in a mouse model of lymphoma that is intrinsically insensitive to ibrutinib but highly expresses PD‐L1 74. Treatment of animals with established tumours by the anti‐PD‐L1 antibody alone resulted in delayed tumour growth and slightly prolonged overall survival, but was not curative. In contrast, the combined treatment with anti‐PD‐L1 and ibrutinib resulted in the cure of approximately 50% of the mice. Tumour‐specific IFN‐*γ* producing T cells were found in the mice treated with the combination of ibrutinib and anti‐PD‐L1 antibody, but not with either agent alone. A similar effect was observed in models of solid tumours lacking BTK and expressing PD‐L1 at very low levels, such as 4T1, a breast cancer model and CT26, a colon cancer model. The authors of the study therefore conclude that ibrutinib is able to potentiate the antitumour effect induced by anti‐PD‐L1 therapy and that this effect is mediated by inhibition of ITK, which provides the rationale for the use of ibrutinib in combination with T cell therapies 74.

Still, the effects of ibrutinib could also be detrimental to antitumour immune responses depending on, for example. NK cell functions. In a preclinical study, an antagonistic effect was observed when ibrutinib was combined with rituximab, an anti‐CD20 antibody widely used in lymphoma treatment 75. Rituximab has various mechanisms of action, for instance antibody‐dependent cell‐mediated cytotoxicity (ADCC). In activated human NK cells, ITK positively regulates Fc receptor (FcR)‐initiated cytotoxicity 52, suggesting that treatment with ibrutinib could impair NK cell‐mediated ADCC. Indeed, the presence of the drug in co‐culture of NK cells with rituximab‐coated lymphoma cells impaired NK cell cytokine secretion in a dose‐dependent manner as well as FcR‐stimulated NK cell degranulation and inhibited NK cell‐mediated cytotoxicity 75. Nevertheless, the direct cytotoxic effect of BTK inhibition on lymphoma cells *in vitro* outweighed the inhibition of NK cell cytotoxicity, making this finding less of a concern for antilymphoma therapy.

### Ibrutinib inhibits BTK and possibly TEC in platelets

Bleeding is one of the common side effects of treatment with ibrutinib. A study including 111 patients with mantle cell lymphoma reported that 17% of the treated patients had contusions and 4.5% had more severe bleedings 76 and a recent 3‐year follow‐up of two other clinical studies reported that 61% of CLL and SLL patients treated with ibrutinib experienced bleeding events 19. The majority of these events were not severe, with contusion and petechiae being the most commonly reported terms. The subcutaneous bleedings observed in ibrutinib‐treated patients are usually not associated with thrombocytopenia 17 indicating a defect in platelet function.

Ibrutinib has been shown to affect platelet function *in vitro*. Platelets from treated patients display an impaired aggregation response to collagen and reduced adhesion to von Willebrand Factor (vWF)‐coated surfaces under high shear conditions, while aggregation responses to other stimuli, such as ADP, thrombin or thromboxane A2 are unaffected 20, 77. The aggregation defect in response to collagen correlates with clinical observations as platelets from patients that experienced bleedings had a significantly lower aggregation response compared to patients without haemostatic complications 20, 77.

The two TFKs expressed by platelets, namely BTK and TEC, are both involved in collagen‐induced platelet activation 78, 79. Binding of collagen to the receptor glycoprotein VI (GPVI) leads to PI3K‐dependent phosphorylation of both molecules independently from each other 65, 79, and they in turn activate PLC*γ*2 65, 78. Autophosphorylation of BTK and phosphorylation of PLC*γ*2 in response to collagen are both reduced in ibrutinib‐treated platelets 20. The collagen response of platelets without functional BTK (i.e. from XLA patients or BTK KO mice) is reduced, but almost normal at high collagen concentrations 65, 78. It is thought that TEC compensates for BTK in these platelets, because platelets from BTK/TEC double KO mice are completely unresponsive to stimulation of the collagen receptor GPVI and show strongly reduced aggregation responses to collagen even at high concentrations 65. The contribution of TEC to collagen signalling in the presence of BTK seems to be minor, as platelets from TEC KO mice have only a slight defect in the aggregation response to collagen 65. Platelet activation by vWF, the other activation pathway that is affected by ibrutinib treatment 20, also involves BTK 80. So far, no role for TEC has been described in this process, but it does not seem to compensate for absent BTK, because no phosphorylation of PLC*γ*2 was found in platelets from BTK‐deficient mice after vWF stimulation, and no stable vWF‐dependent thrombus formation was observed *in vivo* in these mice 81. A recent report showed the importance of BTK and TEC in CLEC‐2 receptor‐mediated platelet activation 82. Activation of platelets by binding of CLEC‐2 to its ligand podoplanin on lymphatic vessel endothelial cells has been found to be a major mechanism for maintaining blood‐lymphatic separation 83. Inhibition of TFKs in murine platelets with ibrutinib abolishes aggregation responses to CLEC‐2 stimulation completely, and embryos of BTK/TEC KO mice exhibit cutaneous oedema with blood‐filled vessels with a lymphatic pattern similar to CLEC‐2‐deficient mice 82. Furthermore, engagement of the fibrinogen receptor integrin *α*_IIb_ *β*_3_ has been reported to induce BTK and TEC phosphorylation 84, 85, indicating that the kinases are not only involved in the initial activation of platelets at sites where the extracellular matrix is exposed to the blood stream, but also in later stages of thrombus formation.

These findings suggest that the platelet aggregation defects observed in ibrutinib‐treated patients are caused by the inhibition of both BTK and TEC in platelets. The impaired responses to collagen and vWF, as well as defective blood‐lymph separation can explain the spontaneous bleedings of these patients. The bleeding events are mostly mild, probably because other activation pathways are not affected by ibrutinib 20, 77, and collagen can still induce very weak responses in the absence of BTK and TEC 65. However, ibrutinib is suspected to have more severe effects in combination with other anticoagulant drugs, and for example, warfarin usage has been excluded from ibrutinib clinical trials 76.

One factor that could possibly play an additional role in the bleeding events under ibrutinib treatment is an effect of the drug on endothelial cells. Endothelial cells express the TEC family kinase BMX 31, 86. BMX has been reported to be involved in angiogenesis and wound healing 55. One could speculate that inhibition of BMX by ibrutinib might affect the integrity of the endothelium. However, mice deficient for BMX have no obvious phenotype and no disturbance of the vascular endothelium 87. The expression in mice is the strongest in big arteries, weaker in small arteries and absent in capillaries 87. The fact that most of the bleeding events in ibrutinib‐treated patients are subcutaneous, that is in places where the vasculature expresses only little or no BMX, makes it less likely that this is an effect of BMX inhibition by ibrutinib.

### Potential Ibrutinib binding to BTK and TEC in osteoclasts

Bone tissue homoeostasis is regulated by bone‐forming osteoblasts and bone‐resorbing osteoclasts. The imbalance of the bone turnover causes various bone disorders 88. Thus, skewing the balance towards osteoclasts leads to the pathologic osteolysis and diseases with low bone mass, while impaired osteoclastic function causes pathologies characterized by a high bone mass, for example osteopetrosis. Both BTK and TEC are expressed in osteoclasts and were shown to play an important role in osteoclastogenesis. In these cells, they are activated by the receptor activator of nuclear factor kappa‐B ligand (RANKL). BTK and TEC kinases link the RANKL and immunoreceptor tyrosine‐based activation motif (ITAM) signals to phosphorylate PLC*γ*2, which leads to stimulation of calcium signalling and activation of NFATc1 (nuclear factor of activated T cells c1), which is the key transcription factor of osteoclastogenesis 89. Mice deficient in both BTK and TEC have a severe osteopetrotic phenotype characterized by increased bone volume and reduced numbers of osteoclasts in the epiphyseal region 64. BTK‐deficient mice have defective osteoclast differentiation 90 but the phenotype of BTK/TEC double KO mice is far more obvious suggesting that TEC has a compensatory function. Furthermore, it was shown that human BTK‐deficient osteoclasts have defective resorption activity *in vitro* but in 20 studied XLA patients abnormal bone metabolism has not been observed, indicating the existence of compensatory mechanisms *in vivo* 91. Thus, it could be speculated that during long‐term use of ibrutinib, binding to both BTK and TEC could have an effect on the bone homoeostasis and possibly lead to pathological conditions due to the lack of bone‐resorbing activity of osteoclasts. To the best of our knowledge, studies of bone density have not been performed as yet in ibrutinib‐treated patients, even if they could be of clinical importance. Nevertheless, ibrutinib was suggested as a treatment option for bone diseases associated with an increased activity of osteoclasts and enhanced bone resorption, as in osteoporosis and rheumatoid arthritis (RA) 92. Ibrutinib's therapeutic effect was shown in an RA mouse model, and it is attributed to the fact that ibrutinib affects both B lymphocytes and inflammatory cells, which are BTK‐expressing effector cells involved in the pathology of arthritis 14, 93.

### Ibrutinib effects on macrophages, neutrophils, dendritic cells and mast cells

TFKs are prominently expressed in haematopoietic cells, where they play crucial roles in lymphocyte development and activation 29. They are also expressed in cells of the myeloid lineage, including monocytes, macrophages, neutrophils, mast cells, erythrocytes and dendritic cells 27. It is known that TFKs play important roles within these cells. Targeting these kinases with ibrutinib will therefore interfere with such functions.

Macrophages contain four TEC family members BTK, TEC, ITK and BMX 27, 94. Several studies show that BTK‐deficient macrophages have impaired phagocytic function and chemotaxis. The murine macrophages that lack BTK have reduced secretion of the proinflammatory cytokines TNF‐*α* and IL‐1*β* after stimulation 27. In monocytes and macrophages, ibrutinib treatment inhibited the BTK auto‐phosphorylation as well as phosphorylation of the BTK substrate PLC*γ*2 and also blocked calcium mobilization and dose dependently inhibited TNF‐*α* and IL‐1*β* production following Fc receptor stimulation 14.

Neutrophils represent the major group of phagocytes. They play a key role in innate immune responses. Human neutrophils express BMX, BTK and TEC 27, 95. These TFKs are crucial for functional activation of neutrophils. Neutrophil adhesion and migration were reduced after treatment with other BTK inhibitors. Following ibrutinib treatment, there was a nearly complete inhibition of neutrophil infiltration into the synovial joints in mice with collagen‐induced arthritis 14.

Dendritic cells (DC) are the most potent antigen‐presenting cells. They express two kinases with an ibrutinib binding site, BTK and BLK (Table 1). It was shown that BTK is essential for human DC function. Signalling through several Toll‐like receptors is impaired in DCs from XLA patients resulting in reduced production of pro‐inflammatory cytokines, and it has been suggested that this may contribute to the susceptibility to infections in these patients. Furthermore, *in vitro* treatment of healthy DCs with ibrutinib resulted in a similar defect 96, 97.

The SRC‐family kinase BLK is expressed in plasmacytoid DCs (pDCs), an interferon type I‐producing subset of DCs that is important for the response to viral infections. In mice with reduced BLK expression, plasma levels of IFN*α* were found to be lower compared to wild type animals 16. Thus, ibrutinib‐mediated activity in DCs could be of clinical relevance for side effects observed in some of the treated patients.

Mast cells play crucial roles in a variety of normal and pathophysiological processes and they are important for the initiation of allergic reactions. In mast cells, BTK, ITK, TEC and RLK/TXK are expressed 27. Among these, the roles for BTK, ITK, and TEC have been investigated. The dependence of mast cells on more than one of TFKs was reported. BTK‐deficient mast cells are defective in degranulation, histamine release and cytokine secretion following FcR stimulation 67. ITK‐deficient mice have been reported to display decreased mast cell degranulation and histamine release. In ITK/BTK double KO mice, these defects were significantly more severe 67. A role for TEC during mast cell activation has also been reported. Studies show that TEC deficient mast cells have impaired production of leukotriene C4 and IL‐4 and that they require both TEC and BTK for the efficient production of TNF‐*α*, IL‐13 and GM‐CSF 66. Thus, it could be assumed that binding of ibrutinib to three of the TFKs and inhibiting their function would have even more severe consequences.

In human cultured mast cells, ibrutinib potently inhibited histamine release following FcR activation. The drug also dose dependently reduced the release of the inflammatory cytokines TNF‐*α* and IL‐8 14, indicating that the drug interferes and impairs mast cell function. Conversely, mast cell inhibition may be clinically advantageous in certain tumour types, in which functional mast cells are required for maintenance of malignant cells 23, 24.

### Ibrutinib and atrial fibrillation

Recently, atrial fibrillation was reported as an adverse effect of ibrutinib treatment, occurring in a few percentages of the patients. The effect was attributed to the binding of ibrutinib to BTK and TEC in the heart and subsequent inhibition of cardioprotective PI3K‐AKT signalling 30. The mentioning that BTK transcripts are expressed in heart tissue is difficult to reconcile as, to our knowledge, this kinase has never been convincingly detected at the protein level in this organ. However, TEC has been detected in rat neonatal cardiomyocytes 98 and in adult mouse cardiac myocytes, where it plays a role in myocardial ischaemia 99, Interestingly, it was also shown that blocking another protein containing an ibrutinib binding site ErbB2/HER2 (Table 1) results in cardiomyocyte dysfunction and reduced heart contractile efficiency. This was observed as an adverse side effect in patients with breast cancer treated by HER2 inhibitors 100. It was also shown that conditional ErbB2/HER2 mutation in ventricular cardiomyocytes leads to impaired cardiac conduction 101 and the protein is required for atrial electrical activity during development 102. Furthermore, another member of the EGFR family, ErbB4/HER4, is also expressed in the myocardiocytes. Heterodimerization of HER4 with HER2 and subsequent downstream signalling, including the PI3K‐AKT pathway, plays a crucial role in heart physiology 103. As described in the previous section, also the ibrutinib‐binding protein BMX was shown to be of importance for the cardiovascular system 53. Thus, there are several possibilities for that ibrutinib's interaction with target proteins other than BTK could cause cardiac dysfunction and atrial fibrillation, and we favour the idea of an off‐BTK target mechanism.

## Conclusions

The therapeutic off‐target effects of ibrutinib studied until now in the haematopoietic system mainly relate to its activity as an ITK inhibitor, which affects helper T cells differentiation, thereby influencing the immune response against tumours and infections, but also potentially harness other immune functions. The final therapeutic outcome of ibrutinib treatment in ‘BTK‐dependent’ diseases will anyhow result from the combination of its on‐target with its off‐target effects. For instance, it may be that clinically relevant adverse manifestations in bone tissue could result from both on‐ and off‐target effects, and that such potential outcomes only appear after long‐term treatment given the rather slow turnover of the bone. Learning more about the influence of ibrutinib on different target proteins, will undoubtedly contribute to the development of strategies for adequate follow‐up of patients and efficient correction of adverse effects. Moreover, there is no doubt that further knowledge of its off‐targets effects likely will pave the way to a broader clinical application of this drug. Thus, such knowledge is useful when considering new indications, where the ‘off‐target’ activity or combination of on‐ and off‐target effects may form the basis for new treatments. To this end, the history of drug development repeatedly teaches us that, what was initially considered a single‐target compound, may later turn out to be a less restricted medicine, but also that an adverse effect may later contribute to the definition of a highly relevant drug target.

## Note

1 For simplification we have used capital letters for all kinases throughout this review.
